# Supplementary figures and images for: Reliable, scalable functional genetics in bloodstream-form Trypanosoma congolense in vitro and in vivo
Source: PLoS Pathog. 2021 Jan 22;17(1):e1009224. doi: 10.1371/journal.ppat.1009224 (PMC7870057; doi:10.1371/journal.ppat.1009224)

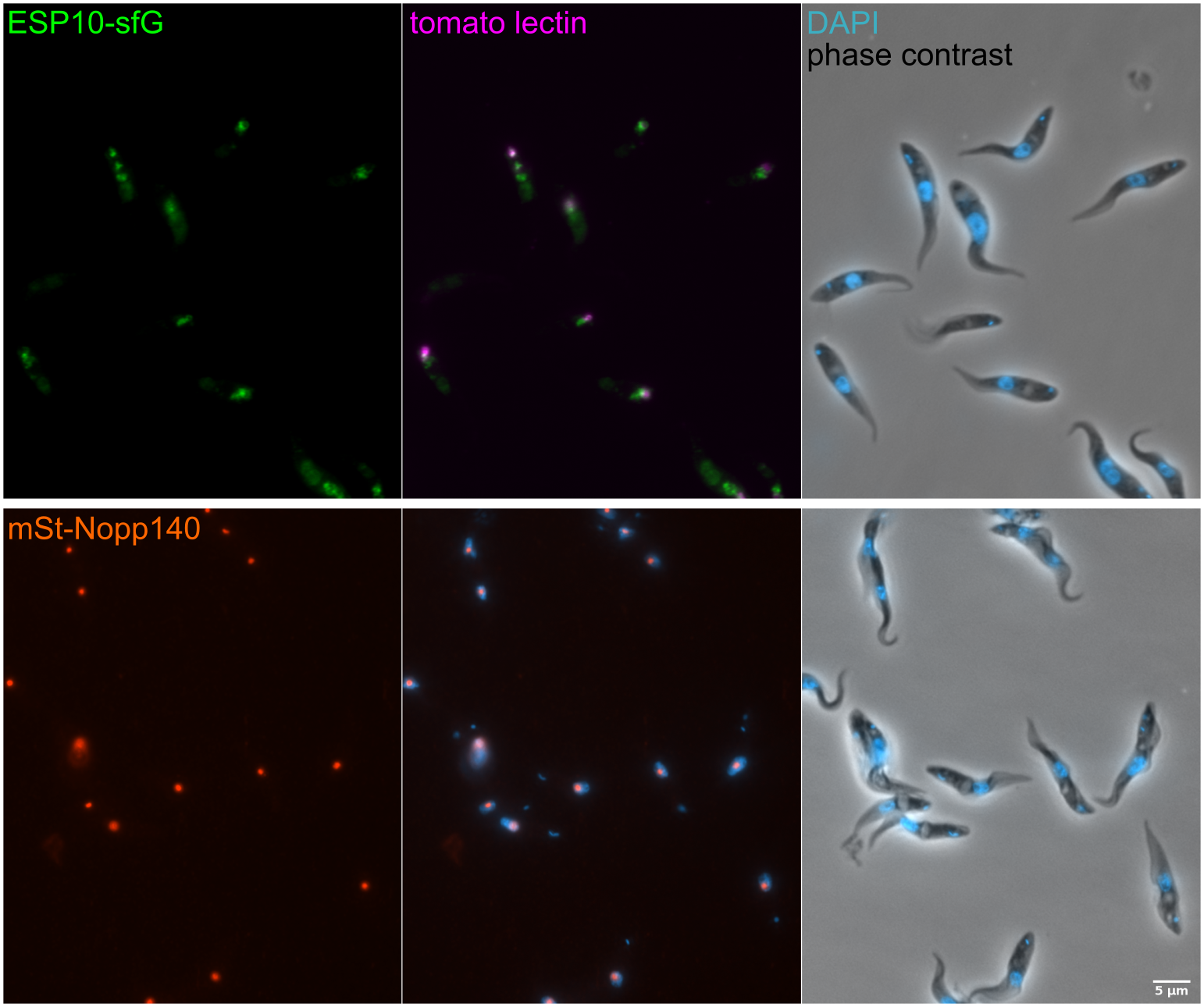

Supplement: S1 Fig — Representative fields of view are shown for cells expressing T. congolense ESP10 C-terminally tagged with superfolder GFP (ESP10-sfG), or Nopp140 N-terminally tagged with mStrawberry (mSt-Nopp140). Native fluorescence from tagged proteins is shown, alongside counter-staining with 4′,6-diamidino-2-phenylindole (DAPI; cyan). ESP10-sfG cells have been additionally stained with AlexaFluor 594-conjugated tomato lectin (TL), to highlight the flagellar pocket and endosomal machinery. (TIF) [file ppat.1009224.s001.tif]

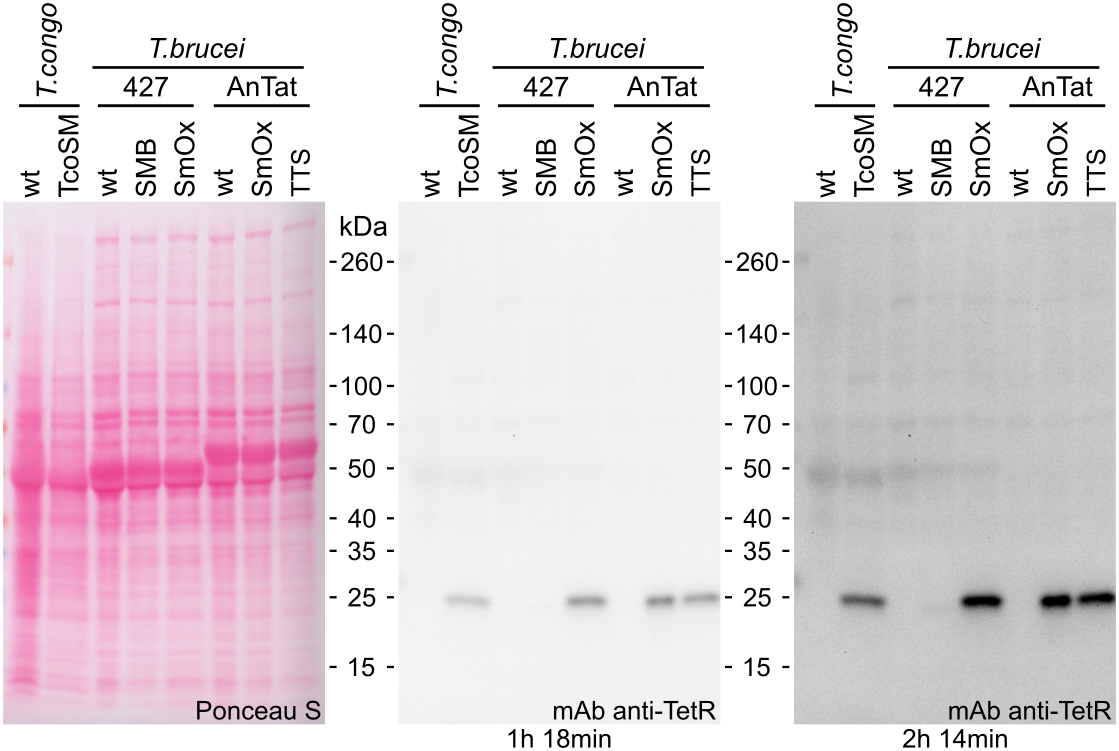

Supplement: S2 Fig — Figure shows full view of immunoblot membrane excerpted in Fig 2D containing transferred whole cell lysates. Ponceau S staining is shown as a control for loading, plus two exposures of the membrane immunoblotted with an anti-TetR monoclonal antibody. ‘wt’ indicates unmodified parental cells. (TIF) [file ppat.1009224.s002.tif]

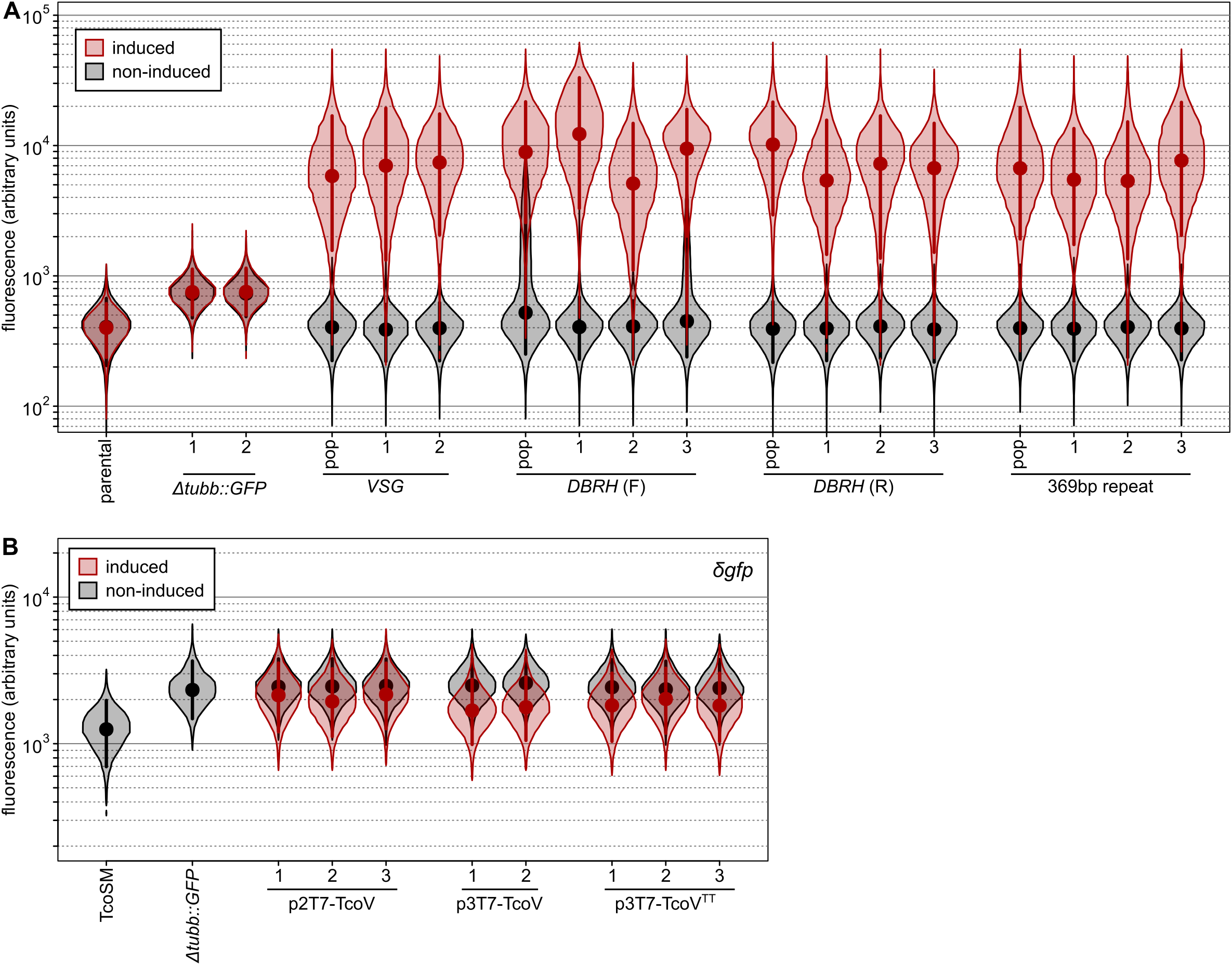

Supplement: S3 Fig — A) Violin plots of green fluorescence of cells expressing GFP from the tubulin locus (Δtubb::GFP) or from inducible minichromosomal loci in 0 (non-induced) or 1 μg ml-1 (induced) tetracycline (see Figs 3A and 4A). B) Violin plots of green fluorescence of cells expressing GFP from the tubulin locus (Δtubb::GFP) in which RNA-interference against GFP had been induced (see Fig 5A and 5B). Median and range of 5% and 95% quantiles are shown by dot and bars, respectively. (TIF) [file ppat.1009224.s003.tif]

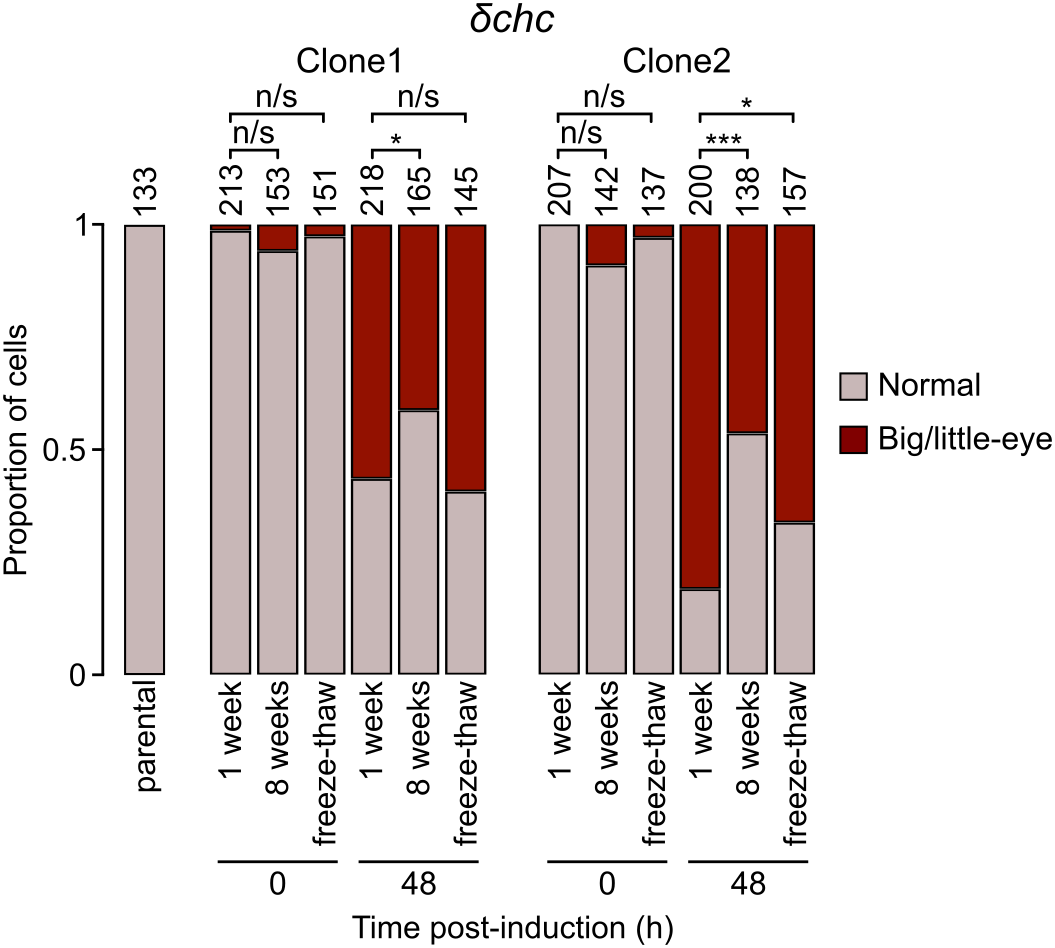

Supplement: S4 Fig — Proportions of cells with clear morphological defect (see Fig 6) are shown for cells with no RNAi construct (parental) and two independent clones of δchc cells following 0 or 48 h induction with tetracycline. Clones had previously been grown in culture for 1 week or 8 weeks following transfection and selection, or frozen after 1 week in culture, stored under liquid nitrogen and then brought back into growth (freeze-thaw). Bonferroni adjusted p-values from proportions test are shown above columns (n/s: not significant; *: p<0.05; ***: p<0.001). (TIF) [file ppat.1009224.s004.tif]

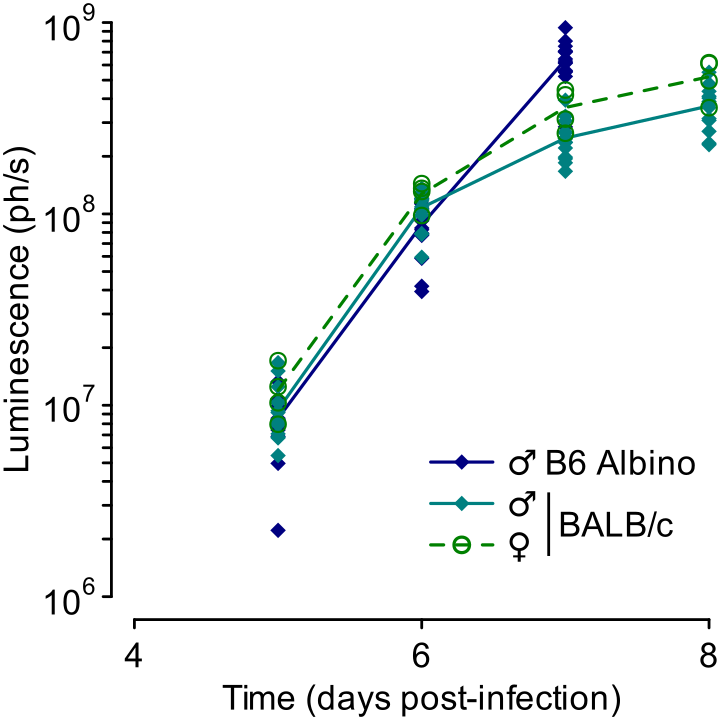

Supplement: S5 Fig — Male or female BALB/c and male B6 Albino (C57BL/6N-TyrcWTSI) were infected with 1000 T. congolense LUC2 cells at time 0 and dynamics monitored by whole-animal bioluminescence. Data from 15 independent infections using male BALB/c and B6 Albino animals, and 4 infections with female BALB/c animals are shown. (TIF) [file ppat.1009224.s005.tif]

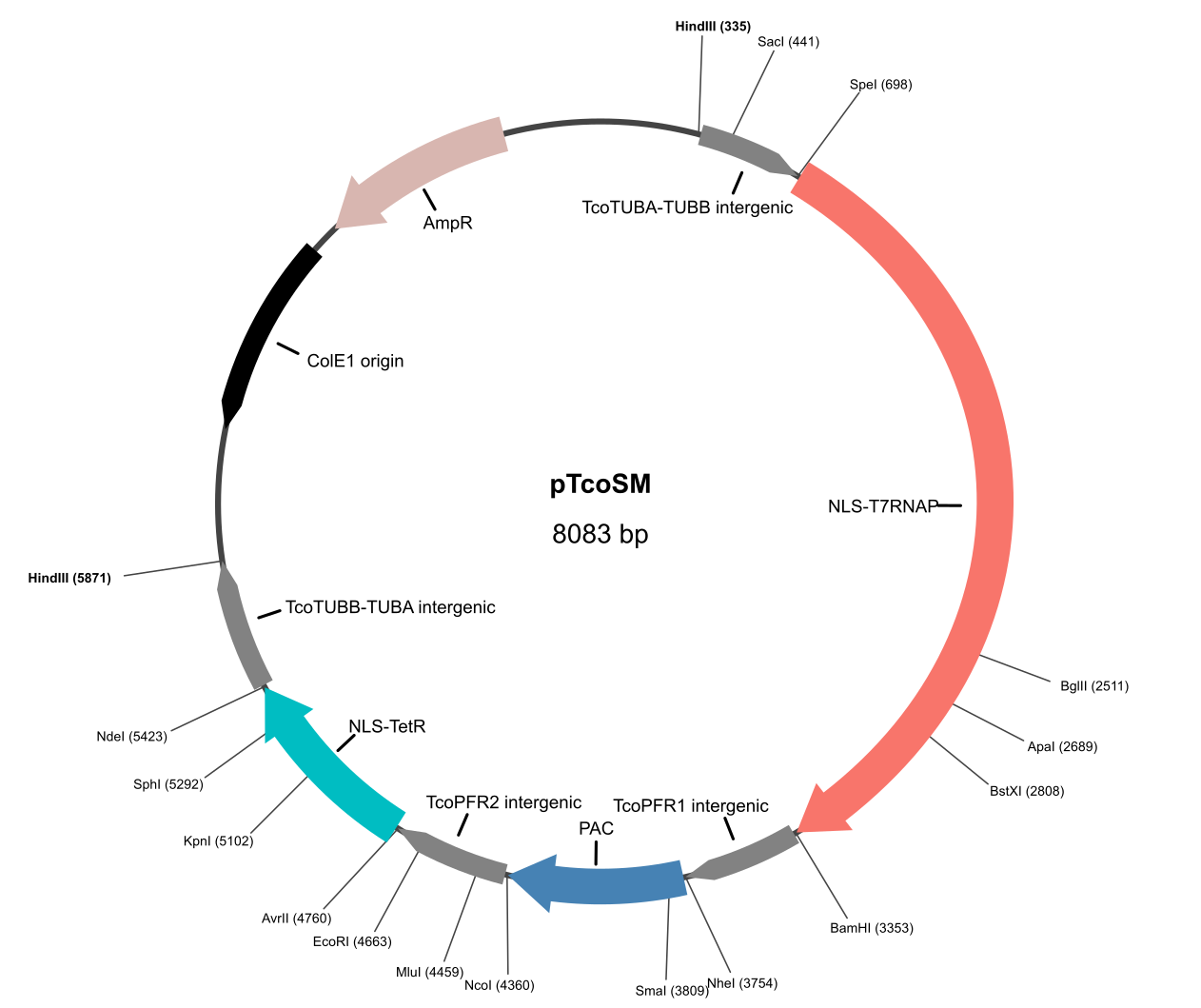

Supplement: S1 Dataset — Annotated sequences are provided in GenBank format and were used to generate graphical maps. (ZIP) [file ppat.1009224.s006.zip › S1_Dataset/pTcoSM.png]

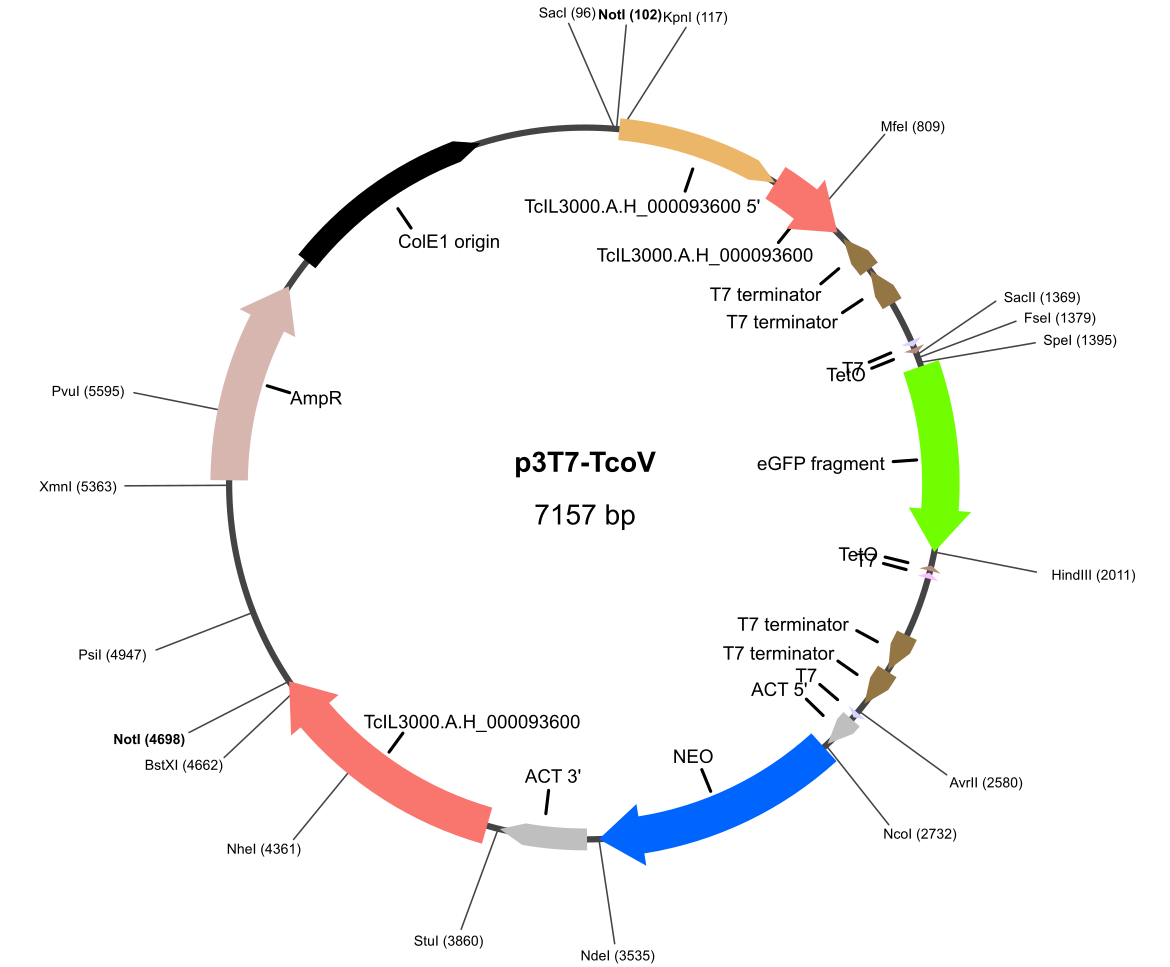

Supplement: S1 Dataset — Annotated sequences are provided in GenBank format and were used to generate graphical maps. (ZIP) [file ppat.1009224.s006.zip › S1_Dataset/p3T7-TcoV.png]

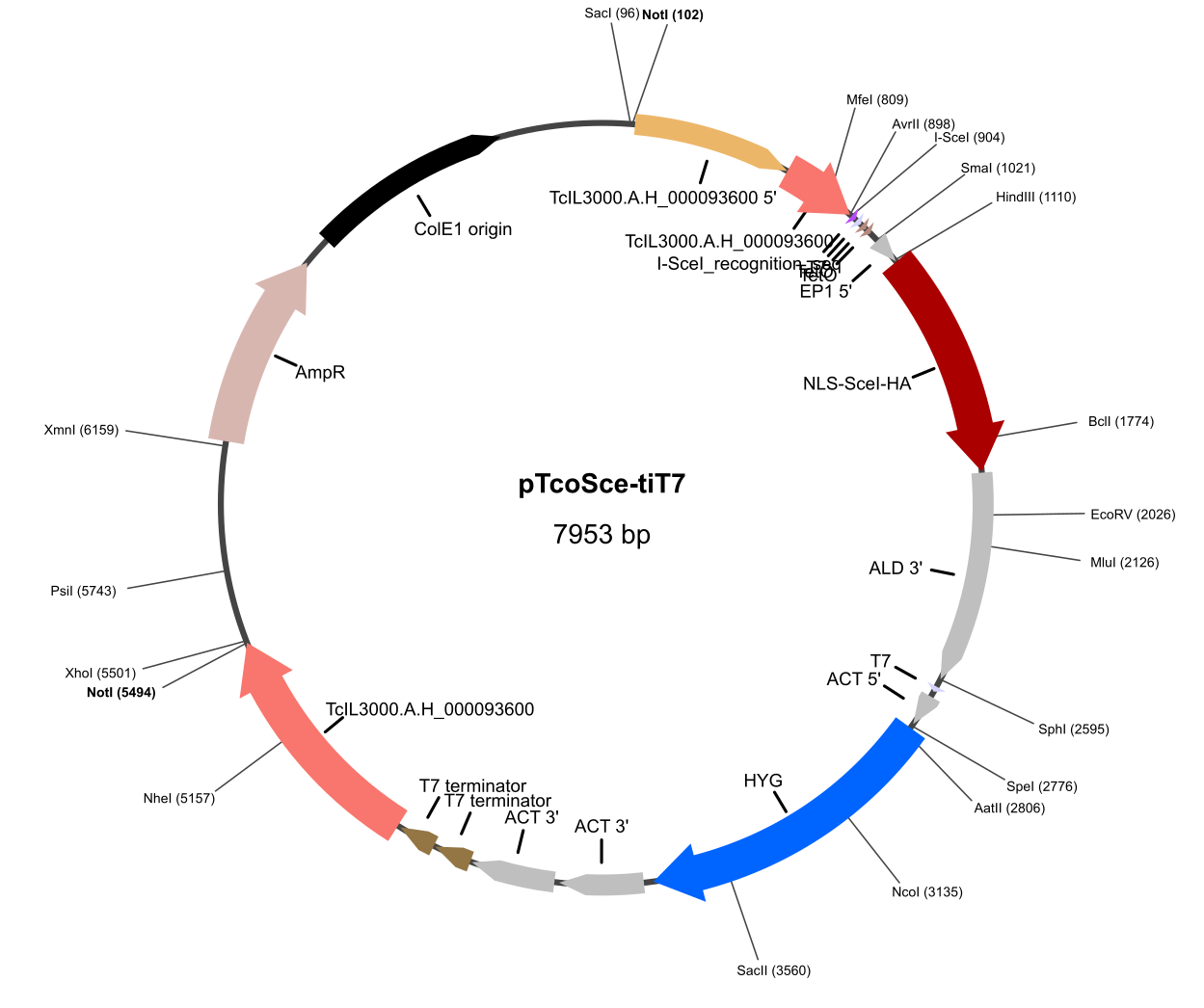

Supplement: S1 Dataset — Annotated sequences are provided in GenBank format and were used to generate graphical maps. (ZIP) [file ppat.1009224.s006.zip › S1_Dataset/pTcoSce-tiT7.png]

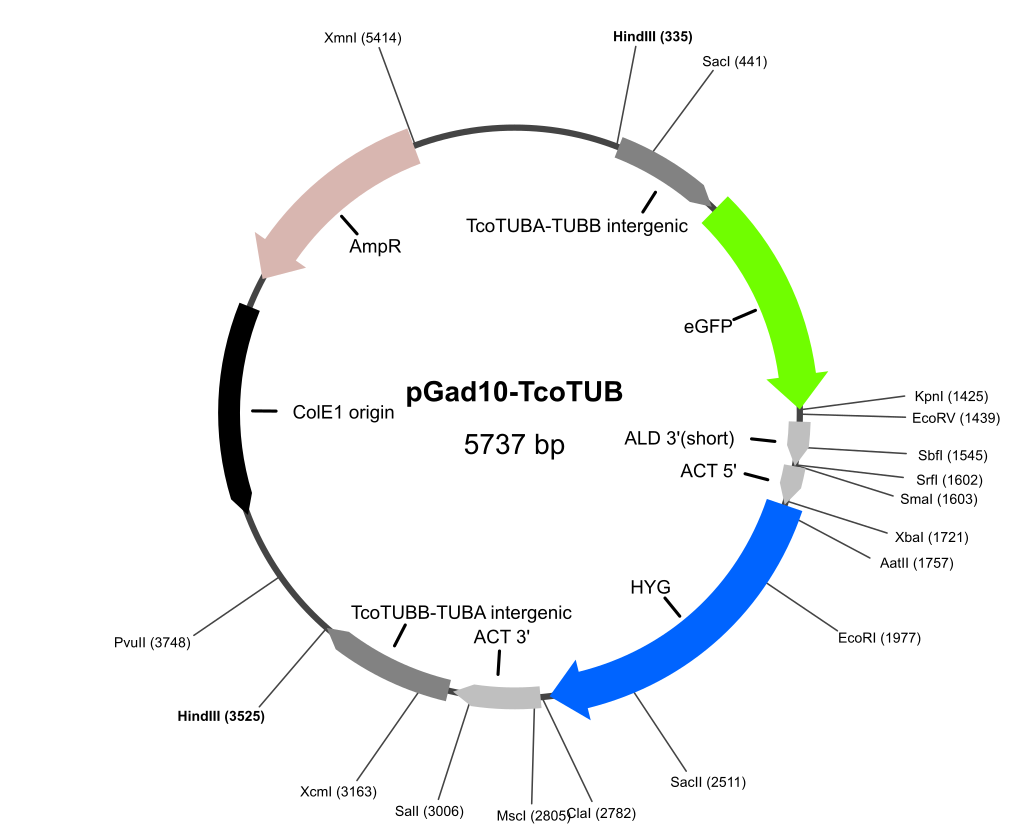

Supplement: S1 Dataset — Annotated sequences are provided in GenBank format and were used to generate graphical maps. (ZIP) [file ppat.1009224.s006.zip › S1_Dataset/pGad10-TcoTUB.png]

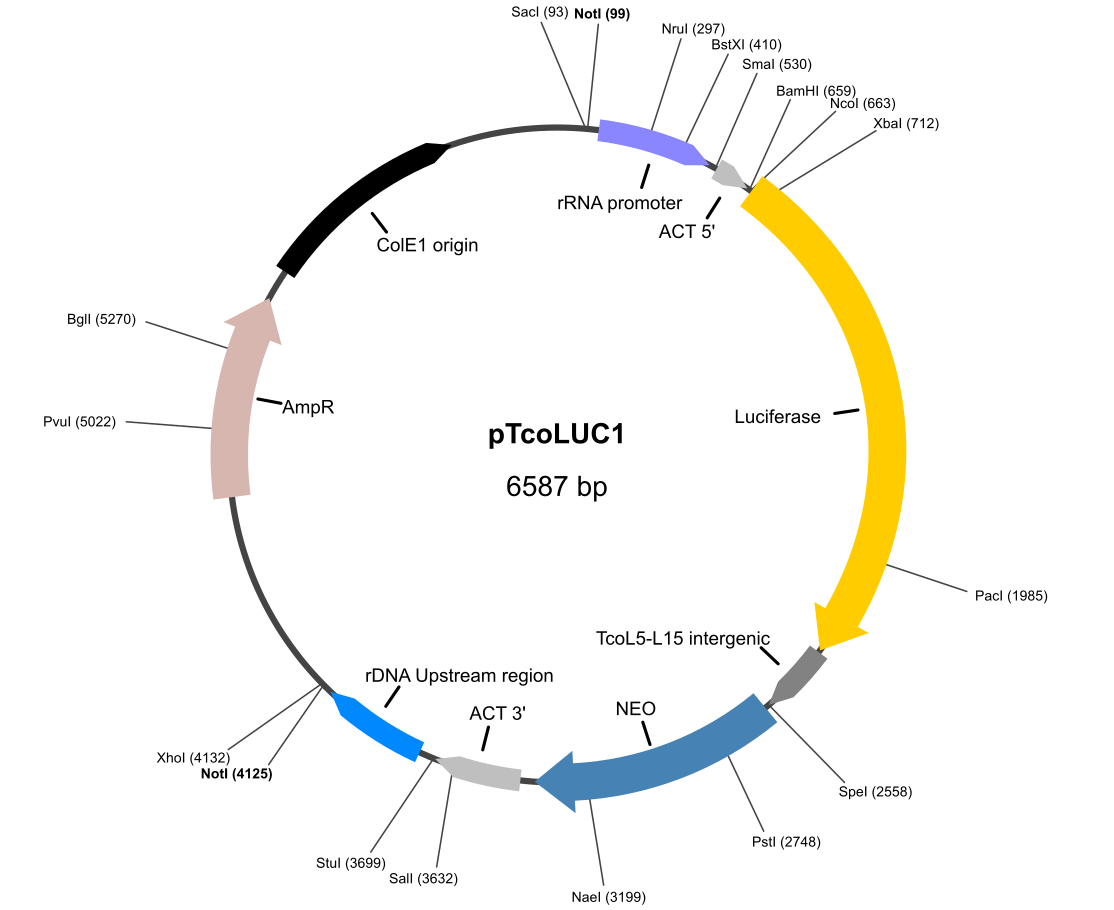

Supplement: S1 Dataset — Annotated sequences are provided in GenBank format and were used to generate graphical maps. (ZIP) [file ppat.1009224.s006.zip › S1_Dataset/pTcoLUC1.png]

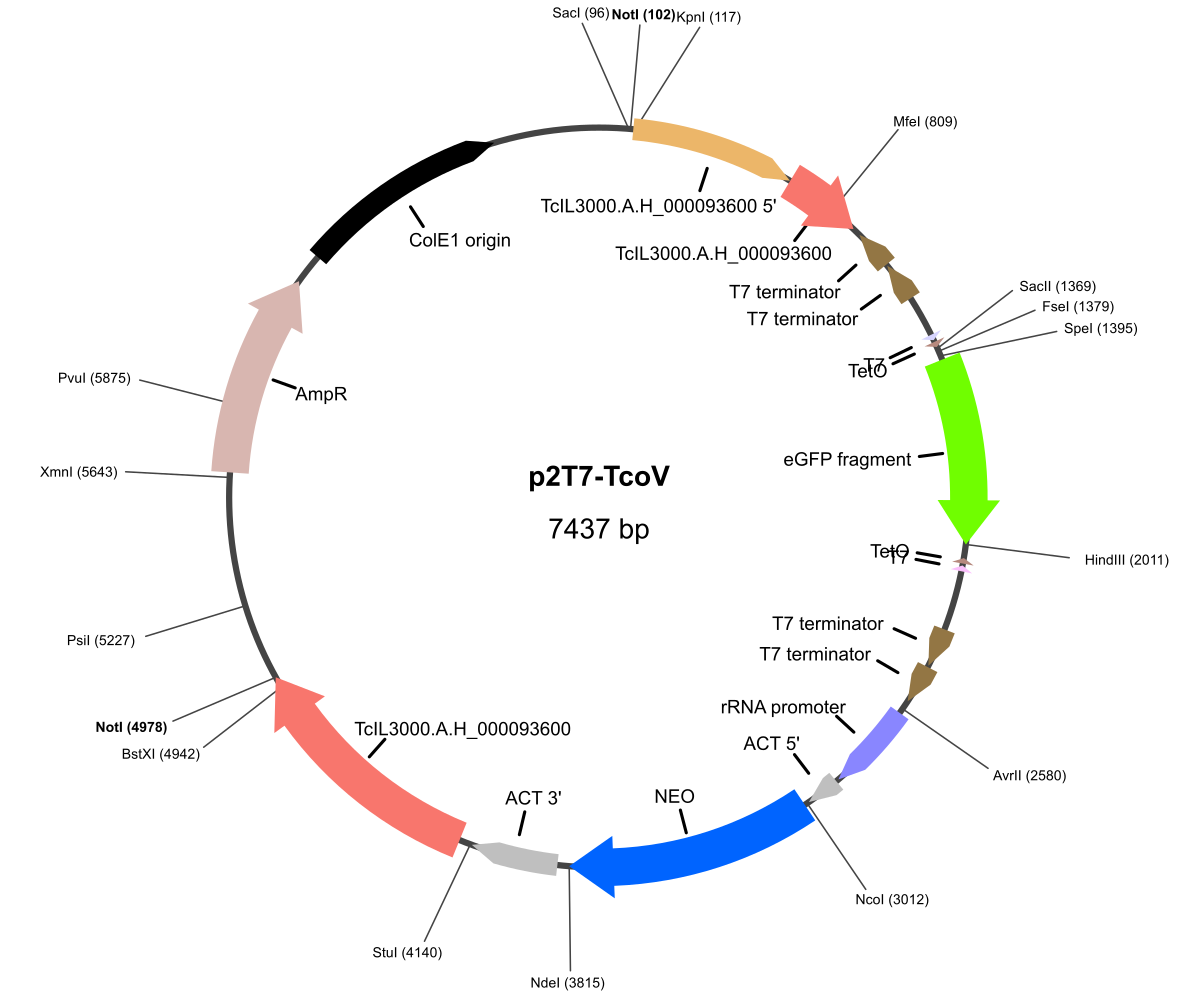

Supplement: S1 Dataset — Annotated sequences are provided in GenBank format and were used to generate graphical maps. (ZIP) [file ppat.1009224.s006.zip › S1_Dataset/p2T7-TcoV.png]

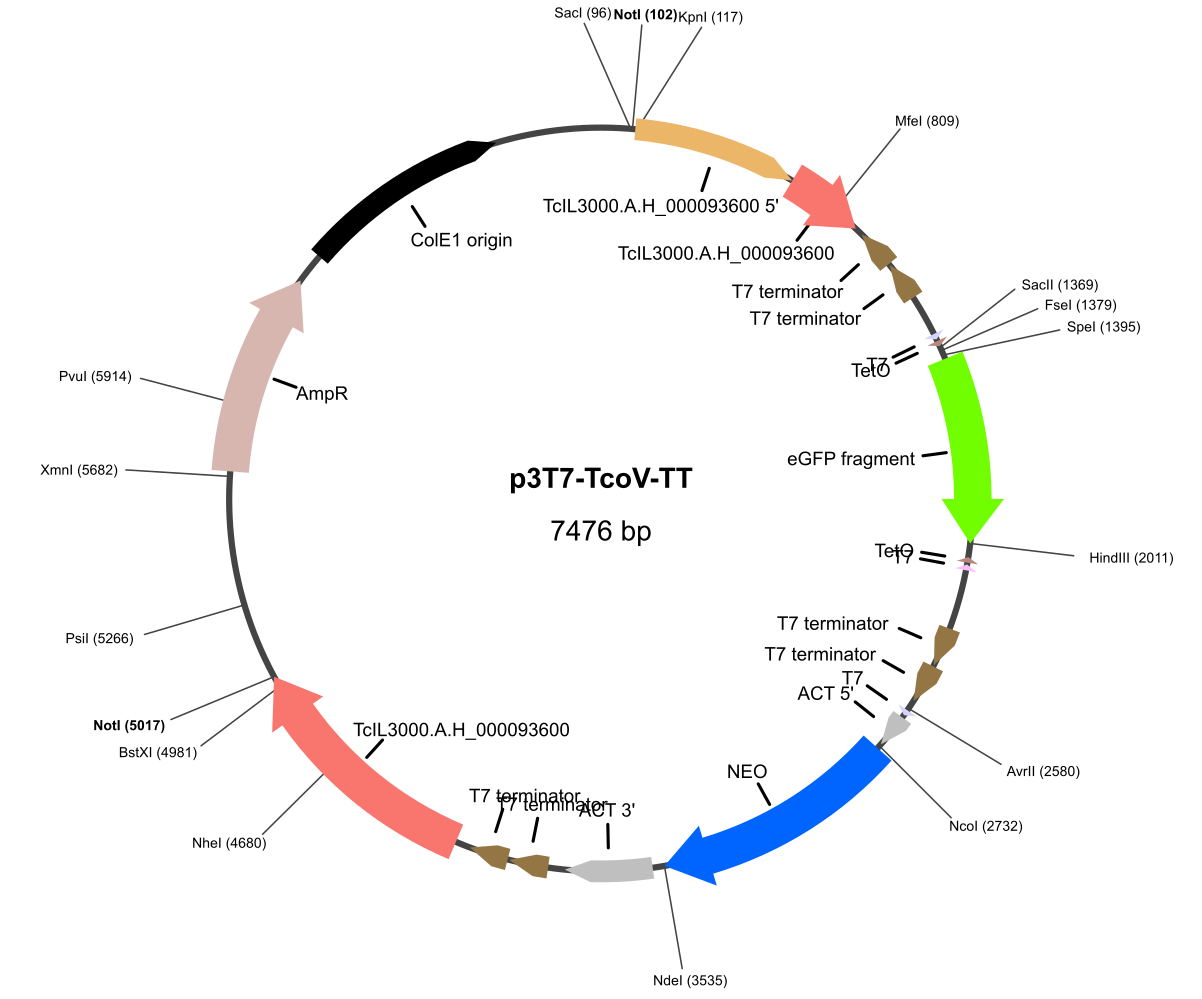

Supplement: S1 Dataset — Annotated sequences are provided in GenBank format and were used to generate graphical maps. (ZIP) [file ppat.1009224.s006.zip › S1_Dataset/p3T7-TcoV-TT.png]

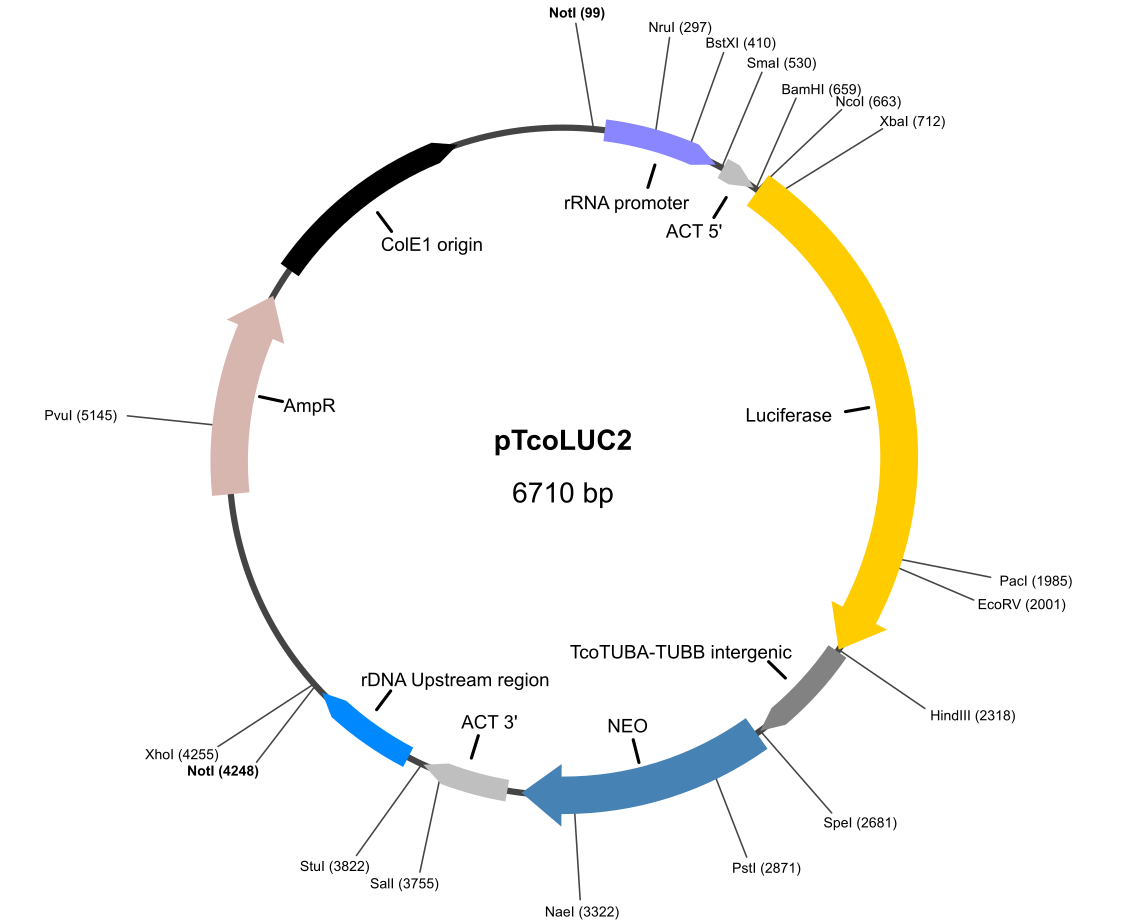

Supplement: S1 Dataset — Annotated sequences are provided in GenBank format and were used to generate graphical maps. (ZIP) [file ppat.1009224.s006.zip › S1_Dataset/pTcoLUC2.png]

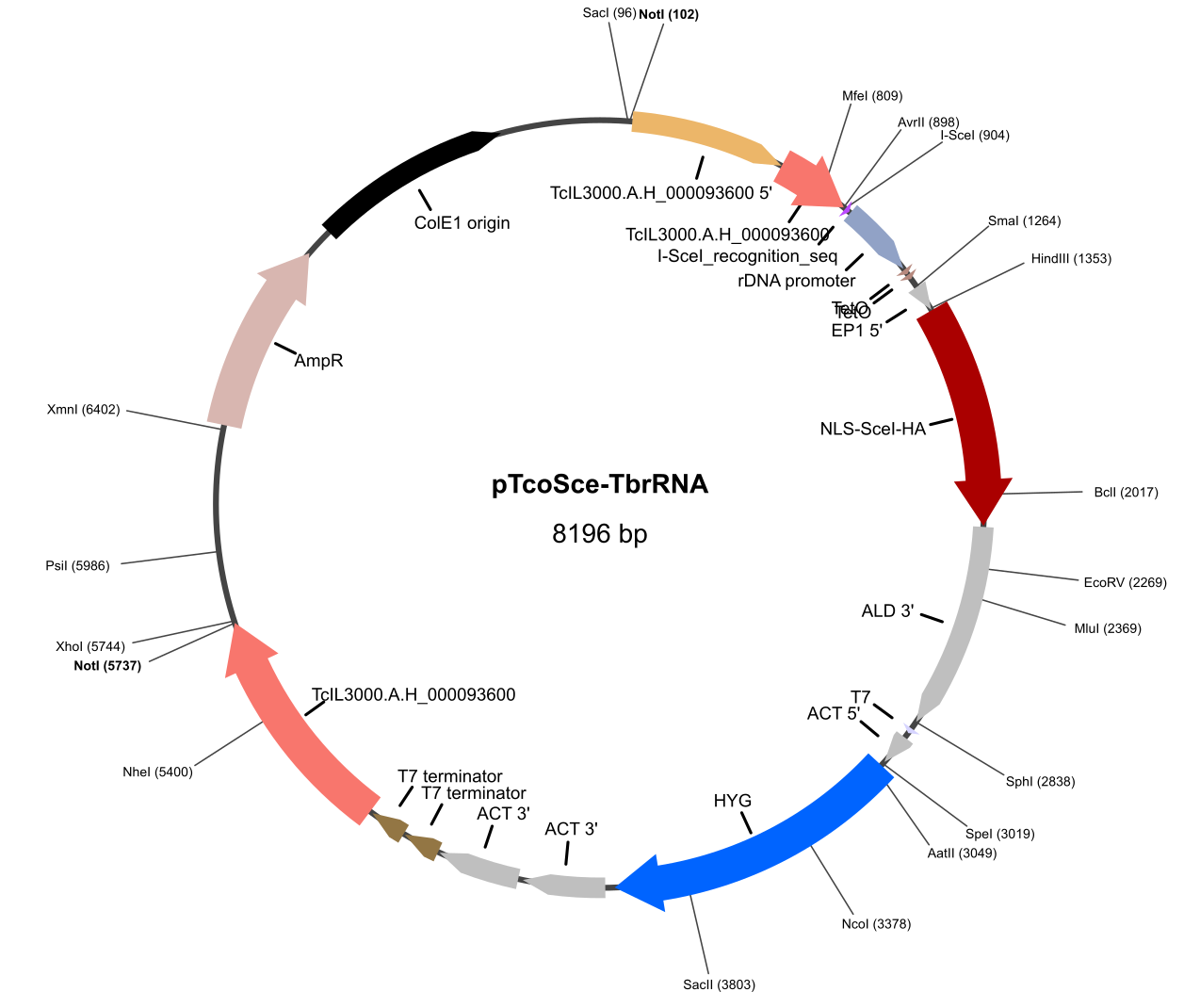

Supplement: S1 Dataset — Annotated sequences are provided in GenBank format and were used to generate graphical maps. (ZIP) [file ppat.1009224.s006.zip › S1_Dataset/pTcoSce-TbrRNA.png]
