# Supplementary material for: Reliable, scalable functional genetics in bloodstream-form Trypanosoma congolense in vitro and in vivo
Source: PLoS Pathog. 2021 Jan 22;17(1):e1009224. doi: 10.1371/journal.ppat.1009224 (PMC7870057; doi:10.1371/journal.ppat.1009224)
Supplement: Attachment — Submitted filename: 201130_Response2PLOSPathogens.pdf [file ppat.1009224.s010.pdf]

Dear Christine and PLoS Pathogens editorial team,

Thank you for the positive reviews of our work "*Reliable, scalable functional genetics in bloodstream-form Trypanosoma congolense in vitro and in vivo*". We are very pleased with the response to our manuscript as we very much agree with the reviewers that work like this should really stimulate research on important trypanosome species other than T. brucei.

Reviewers 1 and 2 had no major issues, but Reviewer 3 and the editorial summary raised a few interesting points that we have addressed below. We have also addressed each of the minor points raised by the Reviewers. In doing so, we have expanded the manuscript by i) creation of 1 new figure and 3 new supplemental figures, ii) inclusion of additional data on transfection, RNAi fragment length, infection dynamics in mouse strains, and expression and conservation of the RNAi machinery, iii) clarification of the text as suggested by the reviewer comments.

In the revised manuscript we have also divided the previous Fig. 3 into two new figures (Fig. 3 and 4). This is only such that the figures can be accommodated in PLoS Path figure size specifications; data contained in the figures are identical to previous.

Thank you for considering our manuscript and we hope the revised manuscript is now suitable for publication in *PLoS Pathogens*.

With best regards,

Catarina Gadelha

Bill Wickstead

PLOS Pathogens: Decision on your submission PPATHOGENS-D-20-02185 -
Please prepare and submit your revised manuscript within 30 days. If you anticipate any delay, please let us know the expected resubmission date by replying to this email.
When you are ready to resubmit, please upload the following:
[1] A letter containing a detailed list of your responses to all review comments, and a description of the changes you have made in the manuscript.

Please see below for detailed responses to individual comments.

Please note while forming your response, if your article is accepted, you may have the opportunity to make the peer review history publicly available. The record will include editor decision letters (with reviews) and your responses to reviewer comments. If eligible, we will contact you to opt in or out

[2] Two versions of the revised manuscript: one with either highlights or tracked changes denoting where the text has been changed; the other a clean version (uploaded as the manuscript file).

Important additional instructions are given below your reviewer comments.

Thank you again for your submission to our journal. We hope that our editorial process has been constructive so far, and we welcome your feedback at any time. Please don't hesitate to contact us if you have any questions or comments.

Sincerely,

Christine Clayton
Associate Editor
PLOS Pathogens

David Sacks
Section Editor
PLOS Pathogens

Kasturi Haldar
Editor-in-Chief
PLOS Pathogens
orcid.org/0000-0001-5065-158X

Michael Malim
Editor-in-Chief
PLOS Pathogens
orcid.org/0000-0002-7699-2064

****

All three reviewers were positive about your paper, although the effectiveness of RNAi is somewhat disappointing. There are however some comments that warrant some extra attention:

a) How long were the siRNAs, and can you get a better knock-down with a longer one?

It's worth noting that knockdown here is not achieved through direct application of exogenous siRNAs, but by production of long dsRNA (which I think are the object of the question here) that are subsequently cut into siRNA by the endogenous machinery. We haven't looked at siRNA length in T. congolense, but their size (~20-27 bp) is conserved between T. brucei and L. braziliensis (and, of course, also much wider), so it's very likely they will be similar here.

The size of the dsRNA fragments in the original manuscript were somewhat buried in the supporting information, so thanks to the Reviewers/Editors for pointing out that we hadn't made this sufficiently clear. All the gene fragments used were in the ~500-800 bp range – specifically designed to allow comparison to a 'normal' head-to-head RNAi performed in T. brucei. It is possible that dsRNAs longer than this or modified in some way to better interact with the RNAi machinery (although it is not clear from work in T. brucei how such a modification might be enacted) might provide better knock-down, but current data would suggest this is unlikely. This, and the comparison to what is known for T. brucei, are important points and in the modified manuscript we have made explicit reference to the sizes in text and figures, as well as some sentences on the interpretation of the data in the Discussion.

b) What is the expression level of the proteins of the RNAi pathway (or at least, levels of mRNA)? Different for different stages?

This, and conservation/divergence of the components, was something that we'd also looked at and we have included these data in the modified manuscript. Unfortunately, there aren't available antibodies that recognise components of T. congolense RNAi. However, mRNA levels suggest the components are expressed very similarly in T. congolense and T. brucei (if anything slightly higher in T. congolense). Of course, mRNA levels (or even protein levels) aren't the same as activity, but the protein sequences in T. congolense show no evidence of loss of function – being no more divergent than one would expect from the position of each species in phylogeny of trypanosomatids. We have included these data in Discussion and in a new Fig. 11.

c) If you differentiate your RNAi lines to procyclic forms, how well (if at all) does the RNAi work? (You must have tried this…)

Although we very much appreciate where this question is coming from, the focus in this work is manipulation of the bloodstream form, as the most relevant for disease modelling and the stage where transgenesis was previously very difficult. There is evidence from the literature that suggests RNAi penetrance is similar in procyclic forms (included in manuscript text). This is also our experience from RNAi in T. congolense procyclic forms (and also epimastigotes), but a full systematic test across multiple genes and clones with quantitation of phenotype + RNA/protein levels has not yet been performed. We feel there is sufficient urgency in getting the systematic analysis of bloodstream form penetrance to the community such that reference to the published work on procyclics is probably sufficient until a systematic analysis is available.

d) Have you tried transiently transfecting dsRNA?

No, we haven't. As noted by Reviewer 3, this might give some additional info on the effectiveness of RNAi in T. congolense – although it presents some non-trivial confounding factors for comparison in terms of total levels of dsRNA received by individual cells and growth rate/RNA stability differences to T. brucei. However, the key comparison we wanted to make here is the effectiveness of RNAi in T. congolense using a system essentially identical to the very common inducible head-to-head system used in T. brucei. Since both systems are driving transcription with the same exogenous polymerase, from single-copy gene fragments of the same size, and inducibly, this seems like the most informative test at this point.

The mechanism behind the lower RNAi penetrance in IL3000 than Lister 427 is definitely of interest for future analysis, but the important findings here are that: 1) inducible RNAi can be easily performed in bloodstream-form T. congolense, 2) an essentially identical system has lower penetrance in T. congolense than T. brucei, but 3) the system can still produce informative phenotypic change. It is worth noting that these would be unchanged by data on transient transfection of dsRNA.

Technical issue: Please state the number of replicates used to obtain error bars or confidence intervals. if it's small then the dot plots are much more informative.

Information on number and type of replicate and also interpretation of bars is now provided explicitly in figure legends.

Reviewer's Responses to Questions

Part I - Summary

Please use this section to discuss strengths/weaknesses of study, novelty/significance, general execution and scholarship.

Reviewer #1: The manuscript by Awuah-Mensah and co-workers describes the successful design and implementation of efficient genetic tools in the protozoan parasite Trypanosoma congolense. The authors build a system that allows for the first time the genetic manipulation of the bloodstream form of this parasite with high efficiency. They also assembled a cell line that allows high-complexity library production that the research community can rapidly make use of. The manuscript is clearly written and it will be of value to the parasitology field. The tools they have developed will allow, among many others, to assess the functional properties of the many proteins whose functions are unknown; to dissect the pathways/genes associated with drug resistance/mode of action or virulence. In this sense, the creation of a bioluminescent cell line is a plus. In addition, the authors show that bloodstream form cells are somehow refractory to

gene silencing via RNAi; however, they do not explore the possible causes nor inspect possible ways to overcome this issue. Still, I am supportive of publication, though make some suggestions for possible improvement of the manuscript.

Reviewer #2: Authors have described successful and better direct genetic modification of bloodstream-form T. congolense IL3000 which provides substantial contribution in trypanosomes research.

Authors describe a parental single marker line that expresses T. congolense-optimized T7 RNA polymerase and Tet repressor and show that minichromosome loci can be used as sites for stable, regulatable transgene expression with low background in non-induced cells. Using these tools, authors describe T. congolense-specific constructs for inducible RNAi and demonstrate knockdown of some essential and nonessential genes. They show that a minichromosomal site can be use to create a stable bloodstream-form line that robustly provides >40,000 independent stable clones per transfection – enabling the production of high complexity libraries of genome-scale. They also show that modified forms of T. congolense are still infectious, create stable high-bioluminescence lines that can be used in models of AAT, and follow the course of infections in mice by in vivo imaging.

The data outlined here focused on bloodstream-form cells, and although the mRNA and proteins efficiency is ~30-50% of parental levels, the tools and methods developed here can be adapted to other replicative stages. The data presented is of high importance to researchers in the trypanosomes field and it opens up the way for gene manipulations in the parasite.

Reviewer #3: In this paper, the authors describe a series of genetic tools that will considerably help the community to study Trypanosoma congolense, a parasite of significant veterinary impact. They have shown that transfection efficiency can be very high when a I-SceI break system is used (40.000 clones per transfection), they generated a single-marker cell-line TcoSM that allows very tight inducible expression of transgenes and created a construct to perform RNAi (p3T7-TcoV).

The manuscript is very clearly written. The experiments are well controlled and interpretations are clear and rigorous.

> *Thanks to the Reviewers for their positive comments on the work. We obviously agree that the work should be of high importance to the trypanosome field – certainly for T. congolense, but also hopefully as a roadmap for other species too.*

Part II – Major Issues: Key Experiments Required for Acceptance

Please use this section to detail the key new experiments or modifications of existing experiments that should be absolutely required to validate study conclusions.

Generally, there should be no more than 3 such required experiments or major modifications for a "Major Revision" recommendation. If more than 3 experiments are necessary to validate the study conclusions, then you are encouraged to recommend "Reject".

Reviewer #1: None noted

Reviewer #2: none

Reviewer #3:

The reduced efficiency of RNAi (to 30-50% of original levels) is somewhat frustrating, given that in T. brucei these values are normally lower (better). Perhaps the AGO/DICER machinery is not as effective in T.

congolense as in T. brucei. Does analysis of transcriptomes of these two species reveal lower transcript levels of RNAi components in T. congolense? The authors could also transfect a dsRNA for tubulin and clathrin heavy chain (transiently) and test the efficiency of depletion. If it is modest as as with the stable genetic system, it would further suggest that RNAi may simply be less efficient in this parasite species. Although it sounds as a "negative result" that RNAi may not be a fantastic tool in T. congolense for non-essential genes, this is an important conclusion, which needs to be published.

> *I think it's important to stress here (also in minor comment #6 from Reviewer 1) that T. congolense are <u>not</u> refractory to RNAi, they're just not as effective at it as T. brucei. We agree that knockdown is not as strong as an ideal system would be – but we should not forget this is also true for RNAi in T. brucei. All RNAi experiments only reduce not eliminate mRNA levels, so phenotypes are always threshold effects. The reduction in T. congolense is lower than in T. brucei, but it is sufficient to produce growth defects when targetting at least some essential genes. It is also perhaps worth mentioning that the Morrison lab hopes to soon publish work in which they have used this system to demonstrate function for non-essential proteins, so I think we would disagree somewhat with the reviewer's pessimism. Definitely not a 'negative result' in our opinion – more a 'limited success'!*
>
> *Re: RNAi machinery conservation and expression. Please see response to note (b) in summary above. This is now addressed in the manuscript text and Fig. 11 of the revised manuscript.*
>
> *Re: transfection of dsRNA. Please see response to note (c) in summary above.*

As the authors mentioned in the Discussion, conditional knock-out might be a better strategy in T. congolense for loss of function studies. It would be important to show that conditional knock-out of tubulin or clathrin heavy chain works very efficiently. This would give the community a good solution for depleting genes of interest.

> *I think whether a 'better' strategy will depend on the parameters of the specific experiment. It would certainly be an alternative where knockdown levels are not sufficient. We haven't tested conditional knockout strategies in this instance, but we have described in our manuscript all the components for such a system – although they would require either another proven drug marker or validated Cre-Lox system to be successfully applied. We think this is outside of the scope of this manuscript and hope the editors will agree that there is sufficient advance in the revised manuscript to warrant publication without this.*

Part III – Minor Issues: Editorial and Data Presentation Modifications

Please use this section for editorial suggestions as well as relatively minor modifications of existing data that would enhance clarity.

Reviewer #1:

1 I found the legends all over the manuscript to be too brief; more details would be beneficial. What 'TL' means in Fig 1B? The manuscript is clearly written by and for people working on trypanosomes, so I consider that adding more details will potentially engage a wider reader audience.

> *Apologies to the reviewer if we weren't sufficiently clear. We have gone through the text and added further details to figure legends.*

2. The transfection protocol described is based on the Amaxa nucleofector which has been a real game changer in the field; however, not every lab has one. It would be beneficial if the author can compare

efficiencies when standard electroporators (BTX, Biorad) are used. If too low, it may save time and money to researchers with low budgets willing to work on T. congolense bloodstream forms.

Beneficial, yes, but we would suggest outside the scope of this manuscript. As the reviewer says, Amaxa nucleofection has given a substantial boost to transfection of kinetoplastids (not just trypanosomes), so it's a real issue that conditions are proprietary to Lonza. It is however now the default used by the community, to the extent that our labs do not possess a BTX or BioRad machine. We can say that electroporation of bloodstream form IL3000 at 3.8 kV/cm in a 10 µF capacitance discharge electroporator (Eppendorf eporator) did not give stable transformants (where it would normally give 10s of procyclic form transformants), but this would require a lot more optimisation to make statements that could be beneficial to labs working on low budgets and is probably outside of the scope of this work.

3. In the same line, the authors claim that its protocol is also useful to transfect PF; however, only one transfection is shown. I understand PF transfection has never been an issue but probably more examples would be clarifying (or rephrase it). In T. brucei for instance, the transfection of PF using Amaxa nucleofector doesn't seem to be more efficient than the old & standard electroporator devices.

We've added data from other procyclic-form transfections to Fig. 1. We've transfected more plasmids than the ones shown here, but mostly with either a different number of cells or with program X-001 (in use before we started the bloodstream-form work) meaning that the direct quantitative comparison cannot be made. All these data suggest essentially equivalent transfection efficiency in procyclics and BSF T. congolense, but we are mindful that this has not been thoroughly investigated. As such, we have been careful that the text only states that "The same preparations of DNA gave very similar numbers of clones when transformed into procyclic form IL3000 cells", which I think reflects the situation accurately.

4. I consider Fig. 2 to be supplemental. As I see it, in every cell line both T7 and tetR are in vastly excess. While tetR dimers must bind to one or two sites, T7pol is highly processive. Independently of this, what is relevant and it is shown later in Fig. 3 is that the assembled cell lines are able to express/repress the transgene in response to the inducer presence/absence and that the fold-change is large enough.

One would certainly hope both polymerase and repressor would be in large molar excess with respect to promoter and operator. However, this is not the same as site occupancy or kinetic availability. Both leakiness and maximal transcription levels are likely influenced by local concentration of T7RNAP and TetR, and increasing these has been suggested to be a potential advantage of the T. brucei SmOx cell lines [Poon et al. Open Biol. (2012) 2:110037]. As such, we think it's important to show that expression of these components is at least as good in TcoSM as in that workhouse of T. brucei biology, SMB. It could certainly be that lower levels would suffice in either organism, but these data show that TcoSM has levels that are sufficient for effective regulation in T. brucei. Given the discussion about RNAi penetrance in particular, we feel these data should remain in the main manuscript.

5. Fig3A. Is the plasmid missing a promoter for the drug resistance or background (uninduced) transcription by T7 is enough for selection? If the latter is true, transcription may not be enough for selection with all drugs; clarify it. In Fig. 3B, it shows the VSG copy number on minichromosomes is "1" but then the authors realize that unfortunately is not the case; please correct the actual number (at least 3/uncertain). Fig. 3D shows the signal range obtained tagging GFP at different loci when induced with 1ug/ml tetracycline. Add a curve showing how the system behaves to different inducer concentrations. Although the chosen representation is informative by showing the total spectrum of values, it doesn't show the proportions of cells displaying such intensities; thus, a standard histogram/violin plot will be much more informative (as in the Suppl). In this sense, I would also prefer Fig. 1 (in situ tagging) to show an histogram/violin plot and not a pic with a single cell (add also a pic where more cells can be seen). Taken together I think not only the fold

change should be considered to select the best locus as heterogeneity/proportion in the signal also affects the decision. This is especially true when the RNAi of diverse genes display such a heterogenous effect on the cell population. In fact, tagging at the DBRH (R) seems to be at the population level the best option (high signal and concentrated); however, the clones then showed larger distributions, that's odd. Any idea?

A lot of smaller points here, so we've addressed separately below:

Fig 3A. This was covered in the text, but the reviewer's comment shows that it was not sufficiently clear and we have modified the text in Results to clarify. Fig. 3A is correct. The plasmid for testing was a single GFP HYG polycistron transcribed by a single tetracycline-responsive T7 promoter (based on the pGad constructs used for regulatable expression from T. brucei minichromosomes [Wickstead et al. (2003) Nucleic Acids Res 31:3993-4000]). No need for selection from background transcription (which is too low): transfectants are selected in the presence of Tet and then Tet removed to assess off-state.

Fig. 3B. We've made Fig. 3B clearer that these numbers refer to copies <u>in the assembly</u>. This is important, as we don't 'know' the copy number of any of these loci – all are only estimates based on the available sequencing – so it would be illegitimate to correct one of the numbers, when the others are still subject to the same limitations. The same principles will apply to the development of other systems at other loci.

Fig. 3D. [now Fig. 4A]. We agree that distributions of cells across intensities is important, and these are shown in S3 Fig. (previously S1) for exactly the reason suggested. However, mean values determine levels expected in population-based assessments such as Western blot, luminescence measurements, qRT-PCR, etc., which are necessary for comparison to data in literature and those presented later in manuscript.

Fig 1. Purpose of this figure was correct cellular localisation rather than total protein levels, which would be very difficult to assess by flow cytometry using native fluorescence. As such, only single representatives were shown (minimal to 'do the job'). To allow comparison of localisation (and levels) across more cells, we have included larger fields of view in a new S1 Fig. We'd be happy to provide more images if required, although these are probably sufficient data for the conclusions being drawn here.

Choice of locus. Fold change was definitely <u>not</u> the only consideration – and 'best' locus will likely vary according to specific application (as it does in T. brucei). In this case, we were looking for a site to introduce constructs (transfection efficiency) that should be highly regulatable (fold change) for transcription running <u>in both directions</u> (F vs. R) and should have low within-clone heterogeneity (distribution of levels) and, ideally, be consistent between clones (site heterogeneity). Data on all of these are presented in Fig. 3, 4 and S3 Fig. In this case, targetting of DBRH (R) would be a poor choice because: i) it gives lower numbers of transfectants, ii) transcription in the forward direction appears to be leaky for at least some DBRH loci, and iii) as noted, the population data for both (F) and (R) orientations suggest there is a fair amount of site heterogeneity in levels. We felt this was probably too much detail to go into in the manuscript, but hope that by providing the data for all the loci, researchers will be able to adapt their systems to fit goals.

6. Regarding the RNAi refractory behaviour of bloodstream forms, there is still plenty room for research. The authors have not checked (or shown) to what extent dsRNA or siRNA are actually produced. It is also known that increasing the dsRNA length (among other factors, mostly thermodynamics), increases the RNAi efficacy; however, it is not indicated how long are the dsRNA utilised here. I suppose the RNAi machinery components and functions are conserved between T. congolense/brucei; please comment on this.

As mentioned in response to Reviewer #3, it is worth noting that T. congolense is <u>not</u> refractory to RNAi, just less effective at it than T. brucei. It is important that the penetrance appears lower

using equivalent approaches, but it is also important that it does work for both essential and non-essential (in all cases tested to date).

Re siRNA/dsRNA. Please see response to note (a) in summary above. Apologies to the Reviewer that we hadn't been sufficiently clear on lengths involved here. All the gene fragments used were in the ~500-800 bp range – specifically designed to allow comparison to a 'normal' head-to-head RNAi performed in T. brucei. These points are now explicitly addressed in the figures and text of the modified manuscript (plus some sentences on the interpretation of the data in the Discussion).

We disagree slightly on the assertion about the effect of increasing length or thermodynamic stability of dsRNA. It is certainly true that in some instances in animal cells, increasing dsRNA length has been shown to increase RNAi efficacy, for other genes, the opposite has been seen (e.g. www.promega.co.uk/resources/pubhub/enotes/rnai-in-drosophila-s2-cells-effect-of-dsrna-size-concentration-and-exposure-time/). Moreover, whether this is true in trypanosomes has not been systematically tested in the literature (sincere apologies if we are overlooking work that does this). In fact, I would argue that most labs performing RNAi in T. brucei freely use any convenient fragment of >250 bp precisely because there is no good evidence that length, GC content, etc. make a substantial difference. Here, we feel the most important point for the application of the technology is the functional comparison to T. brucei.

Re: RNAi machinery conservation and expression. Please see response to note (b) in summary above. This is now addressed in the manuscript text and Fig. 11 of the revised manuscript.

7. In Fig. 7 the authors calculate the transfection efficiency using a single plasmid. The use of a library where plasmids will have different sizes, GC content and so on may affect this number; please comment on this. Fig 7C; I understand the legend is missing the integration of the tub RNAi construct. I had to go through these lines several times to understand what you did, please rephrase it.

Exactly right – especially with respect to plasmid targetting sequence. This is precisely why we have tested increase in efficiency due to double-strand break induction (Fig. 8 and 9; previously 7 and 8) with not just a single plasmid, but a single preparation of a single plasmid - eliminating as much potential variation in other parameters as possible. However, in the manuscript we developed a set of targetting tools for this locus, <u>all of which contain identical targetting sequence</u>. In this case, we have tested insertion of a 'typical' sized RNAi construct, which would differ only in the gene fragment for a different knockdown, which would be expected to have identical efficiency for insertion. It is worth noting that the RNAi plasmid is not even one of the most efficient plasmids for integration, so it is likely that other plasmids could be integrated with even greater efficiency with double-strand break, but it was essential for the application of this method to produce, for example, RIT-Seq-like libraries that an RNAi plasmid against an essential gene was used.

Thanks to the Reviewer for the comment about legend clarity. We have modified the legend, which we hope will make it clearer for the reader.

8. Please comment about the quality of the reference genome IL3000. A well curated genome is vital to perform molecular biology. In this sense, have the authors tried to modify others T. congolense strains? Are they expected to be easily transfected?

The latest genome assembly for IL3000 ("IL3000_2019") is a de novo assembly based on PacBio reads. As such, it has much better coverage of repeats and areas that differ greatly in organisation to T. brucei, but some gene models suffer from the higher proportion of technical INDELs that PacBio sequencing produces compared to Illumina. The former was absolutely essential for this work as it allows the identification of minichromosomal sequences that will never appear in a genome scaffolded on T. brucei sequence. The latter is an inconvenience,

although generally not a major issue for most sequencing work, and gene model differences are generally obvious by comparison to the earlier short-read assemblies. Curation is improving as interest in the organism grows, and of course should continue to improve. In the end, it's the genome assembly we have got, and enough of a resource to catalyse a huge amount of work.

We would love to modify other T. congolense strains, and additional strains (particularly representing forest or Kenya Coast/Kilifi groups) will be a really important future development. However, at present there are essentially no other strains that can be grown in culture as stable bloodstream forms. This is undoubtedly a restriction to current T. congolense work (although it is perhaps worth reflecting on how little exploration of 'strain space' occurs even for T. brucei, where many strains can be readily grown). More importantly, however, this work shows that as soon as another strain is established in culture, we should be able to very quickly raise it to a tool for molecular genetics.

9. How stable is the TcoTTS cell line? for how long have the authors grown/transfecting them without affecting efficiency? Such a stable cell line will save quite a lot of effort to the research community and should be an inspiration for the T. brucei one.

We fully agree. Stability of TcoTTS with respect to growth and storage is a really important advantage to the technology. This was covered in Fig. 7B (now Fig. 8B), but we've made the text around stability clearer.

Reviewer #2:

1. Introduction

The introduction/ literature is very good except that the information that "T. congolense LIKELY causes the majority of disease in sub-Saharan cattle (2,3), as well as making up a substantial proportion of infections (4)" cannot be entirely true as the predominance of a particular species may vary from place to place. The use of "MAY" should be more appropriate.

Modified as suggested.

2. Results

a. In Figure 2D - the wells as seen in the PonceauS does not properly correspond to the bands seen on the western blot. It looks like some parts were cut off (WT) and the TcoSM band looks stretched out as if the sample was loaded in a thinner well.

Not a thinner well, but of course samples in an SDS-PAGE gels do sometimes run slightly unevenly. It's worth noting that as indicated in the figure, the Ponceau S image covers a different area of the membrane (containing VSG) to allow easier comparison of protein levels. To allow the full data to be assessed, we've included full views of the membrane and additional exposures of the immunoblot signal as Supplemental data (S2 Fig).

b. In Figure 3C – Authors have indicated that "All lines were captured and processed equally, except that GFP signal however at a closer look it seems some of the cells looks relatively smaller than the others e.g. in DBRH (F) and (R) and 369bp repeat. why is this so? It will be useful to clarify the differences here.

I have to admit that we can't see the size difference the reviewer is alluding to here. The statement about processing was specifically to allow comparison of fluorescence levels, as one would hope that it would always apply to image sizes. In this case, all fields in the figure are 61.92 x 51.60µm captured with the same 100x objective and camera, and are displayed at

native pixel ratio (1:1). The microscope used has no secondary objective, so any difference due to use of an inappropriate objective (40x) would be immediately obvious. Reviewing the microscopy, we can find no evidence of systematic cell size differences between the cell lines – either across all images captured or in the representative images used in the figure. We'd be happy to supply original images for editorial review, if the reviewer felt it necessary.

c. Figure 4 – it will be useful to represent the % knockdown (quantification) below the upper gel 4D. It was not clear the criteria for choosing 48h induction in some cases and 72h inductions in other situations as even a longer RNAi had a relatively lower reduction compared to T. brucei (For example, in Figure 4D and E).

I see the reviewer's intent here, but there's a potential to confuse 'proper' quantitative methods (flow cytometry, qPCR) with semi-quantitative methods (chemiluminescence). Values on the figure are given for the fully quantitative methods only. As such, we'd prefer to only refer to in the text ("<10% of parental" and "~30%", respectively).

For ESP14, we have previously shown that maximal knockdown is achieved by 24h in T. brucei. For comparison, maximal knockdown of T. congolense (indistinguishable at 48h or 72h) was selected. Knockdown in all cases studied seems to get no better after 48h (if anything slightly worse), so time points reflect this. However, note that 6 genes are followed at either mRNA level (Fig. 5F) or phenotypically (Fig. 6B) across multiple time points.

d. Also the RNAi against a further 5 endogenous genes (PPDK, TcIL3000.A.H_000922100; CHC, TcIL3000.A.H_000768200; FBPase, TcIL3000.A.H_000671500; FH1, TcIL3000.A.H_000909500; PEPCK, TcIL3000.A.H_000300300) consistently reduced mRNA levels, but only to 30-60% of parental or non-induced levels (Fig. 4E,F). However, there is increase in mRNA levels after 48hours for all transcripts 4F. Authors should clarify this in the time course experiment.

Agree – see also phenotype penetrance in Fig. 6. We had felt this was clear, but comments from review suggest insufficiently so. We have now added an explicit mention of the interpretation to the Results for clarity.

e. Figure 5 – is there a special reason/s for having the two different pictures at 48h lower and upper pictures? Do they give the same information? 5A. What are the error bars on 5C? how many independent experiments per cell line?

Only reason was to show more cells to give representation of heterogeneity. We will remove fields of view if requested, but here we would agree with Reviewer 1 that more cells are necessary to give good representation. Error bars in 5C (and 2B) were from technical replicates, and have been removed in the interests of clarity (only one fully independent estimate per time point, but across multiple time points and 2 clones).

Methods

In writing references two systems are written. While most of the references are given by numbers, Line 435 has - TcPCF3 (14). Bloodstream-form T. brucei SMB cells (Wirtz et al., 1999) were grown in HMI-9 medium supplemented with 15% fetal bovine serum at 37ÅãC and 5% CO_2 (43).

Thanks to the Reviewer for spotting this error in citation formatting. Corrected in the modified manuscript.

References

While some of the references have the month and day this is omitted in other (2, 5, 9, 12 etc).

Again thanks for spotting. This is an inconsistency in the Vancouver style specification in our reference manager. Corrected in modified manuscript.

Reviewer #3:

To test the virulence of the transgenic T. congolense lines, authors infected BALB/c mice. Could the authors explain why they chose this strain of mice, instead of C57BL/6 mice?

For compatibility with infection monitoring using in vivo imaging system, it is necessary to use albino animals. BALB/c were selected to develop the acute infection model as an albino strain with well-studied immunity. However, infection dynamics are very similar in B6 Albino (natural albino mutant of C57BL/6N). We have added these data and information on effect of mouse gender as S5 Fig.

Do you want your identity to be public for this peer review? For information about this choice, including consent withdrawal, please see our Privacy Policy.

Reviewer #1: **Yes:** Esteban D. Erben

Reviewer #2: No

Reviewer #3: No

Figure Files:

While revising your submission, please upload your figure files to the Preflight Analysis and Conversion Engine (PACE) digital diagnostic tool, https://pacev2.apexcovantage.com. PACE helps ensure that figures meet PLOS requirements. To use PACE, you must first register as a user. Then, login and navigate to the UPLOAD tab, where you will find detailed instructions on how to use the tool. If you encounter any issues or have any questions when using PACE, please email us at figures@plos.org.

Data Requirements:

Please note that, as a condition of publication, PLOS' data policy requires that you make available all data used to draw the conclusions outlined in your manuscript. Data must be deposited in an appropriate repository, included within the body of the manuscript, or uploaded as supporting information. This includes all numerical values that were used to generate graphs, histograms etc.. For an example see here: http://www.plosbiology.org/article/info%3Adoi%2F10.1371%2Fjournal.pbio.1001908#s5.

Reproducibility:

To enhance the reproducibility of your results, PLOS recommends that you deposit laboratory protocols in protocols.io, where a protocol can be assigned its own identifier (DOI) such that it can be cited independently in the future. For instructions see http://journals.plos.org/plospathogens/s/submission-guidelines#loc-materials-and-methods

---

In compliance with data protection regulations, you may request that we remove your personal registration details at any time. (Remove my information/details). Please contact the publication office if you have any questions.
